# LOSS OF HYPOXIA SIGNALING LIMITS SKELETAL MUSCLE RESPONSE TO AEROBIC EXERCISE IN AGING

**DOI:** 10.1093/geroni/igz038.342

**Published:** 2019-11-08

**Authors:** Yori Endo, Bin Li, Kodi Udeh, Adriana C Panayi, Ron Neppl, Indranil Sinha

**Affiliations:** 1 Harvard Medical School, Boston, Massachusetts, United States; 2 Brigham and Women’s Hospital, Division of Plastic Surgery, Boston, Massachusetts, United States; 3 Brigham and Women’s Hospital, Harvard Medical School, Boston, Massachusetts, United States; 4 Brigham and Women's Hospital, Department of Orthopaedic Surgery, Boston, Massachusetts, United States

## Abstract

Skeletal muscle function declines with aging. Physical activity improves muscle function, but may require upregulation of the hypoxia signalling pathway. In this study, we examine the effect of exercise on muscle function in young and old mice and explore the role of hypoxia pathway in mediating exercise-induced functional improvement. Young (3 months) and old (22-24 months) mice underwent either aerobic exercise training on a treadmill for 8 weeks (40 minutes per run, 3 times weekly, at 8 meters/min at a 15 degree inclination) or no exercise as a control. Maximum running speed and distance were measured pre- and post-exercise. Following training, there was a significant increase in both maximal speed (20%, p<0.05) and distance (2.5 fold) running in young mice which exercised versus young control (p<0.01). Old mice following exercise training did exhibit any improvement versus the old control group. Notably, protein levels of aryl hydrocarbon receptor nuclear translocator (ARNT), a critical regulator of hypoxia signalling, was 4.7-fold lower in the muscle of old mice after exercise as compared to young (p<0.01). To evaluate further whether loss of hypoxia signaling limits response to exercise, we developed a mouse with an inducible, skeletal muscle specific ARNT knockout (ARNT mKO). ARNT mKO mice exhibited no improvement in either maximal running speed or distance following exercise training. In contrast, littermate controls, with normal ARNT levels, exhibited improvement in both maximal speed and distance running following exercise. Our results suggest that an aging-associated decline ARNT and hypoxia signalling may limit aerobic exercise benefits.

